# Gambling expenditure by game type among weekly gamblers in Finland

**DOI:** 10.1186/s12889-018-5613-4

**Published:** 2018-06-05

**Authors:** Anne H. Salonen, Jukka Kontto, Riku Perhoniemi, Hannu Alho, Sari Castrén

**Affiliations:** 1 Alcohol, Drugs and Addictions Unit, National Institute for Health and Welfare, P.O. Box 30, FI-00271 Helsinki, Finland; 2 Faculty of Health Sciences, University of Eastern Finland, Kuopio, Finland; 3 Public Health Evaluation and Projection Unit, National Institute for Health and Welfare, Helsinki, Finland; 4 Abdominal Center, University and University Hospital of Helsinki, Helsinki, Finland; 5 Department of Psychology and Speech-Language Pathology, Faculty of Social Sciences, University of Turku, Turku, Finland

**Keywords:** Cross-sectional, Game type, Gambling expenditure, Net income, Population study, Relative gambling expenditure

## Abstract

**Background:**

Excessive expenditure and financial harms are core features of problem gambling. There are various forms of gambling and their nature varies. The aim was to measure gambling expenditure by game type while controlling for demographics and other gambling participation factors. A further aim was to find out how each game type was associated with gambling expenditure when the number of game types played is adjusted for.

**Methods:**

Using data from the 2015 Finnish Gambling survey on adult gamblers (*n* = 3555), multiple log-linear regression was used to examine the effects of demographics, gambling participation, and engaging in different game types on weekly gambling expenditure (WGE) and relative gambling expenditure (RGE).

**Results:**

Male gender, lower education level, higher gambling frequency and higher number of game types increased both WGE and RGE, while younger age decreased WGE but increased RGE. Furthermore, seven specific game types increased both WGE and RGE. Weekly horse race betting and non-monopoly gambling had the strongest increasing effect on expenditure. Betting games and online poker were associated with higher expenditure even when they were played less often than weekly. Among weekly gamblers the highest mean WGE was recorded for those who played non-monopoly games (146.84 €/week), online poker (59.61 €/week), scratch games (51.77 €/week) and horse race betting (48.67 €/week). Those who played only 1–2 game types a week had the highest mean WGE and RGE on horse race betting and other betting games.

**Conclusions:**

It seems that overall gambling frequency is the strongest indicator of high gambling expenditure. Our results showed that different game types had different effect sizes on gambling expenditure. Weekly gambling on horse races and non-monopoly games had the greatest increasing effect on expenditure. However, different game types also varied based on their popularity. The extent of potential harms caused by high expenditure therefore also varies on the population level. Based on our results, future prevention and harm minimization efforts should be tailored to different game types for greater effectiveness.

## Background

Early research into the adverse consequences of gambling was focused on the presence of pathological or problem gambling, but recently it has become commonplace to take a broader view on gambling harm [1, 2]. Some game types, for example, slot machine gambling, casino games, poker, betting games, bingo and/or scratch games have been associated with gambling-related problems (e.g. [3–8]). On the other hand, it has been suggested, that some game types may be more like indicators of unhealthy gambling involvement, rather than critical factors associated with gambling-related problems [9, 10]. Gambling expenditure, one of the indicators of unhealthy gambling involvement, shows the strongest association with gambling-related harm as many of the negative impacts of excessive gambling are due to financial problems [1, 2, 11–14]. Despite this association, gambling problem or even gambling-related financial harm are not synonymous with excessive expenditure [15, 16]. For harm prevention and minimization purposes it is essential that we build our understanding of different game types and associated harms. There is as yet very little research on gambling expenditure by game type.

Finland has one of the highest per capita gambling expenditure rates in Europe [17]. For research purposes gambling expenditure is usually assessed by questions concerning wins and losses, or most typically by direct questions on spending; the latter is the most common way [18]. However, it has been suggested that in order to gain a clearer picture of gambling-related harm, gambling expenditure should be examined in relation to the gambler’s net income [2]. We use the dual measures of weekly gambling expenditure and gambling expenditure in relation to the gambler’s net income.

Gambling expenditure is higher among men than women [19–23]. Furthermore, low education and unemployment are associated with higher gambling expenditure [20, 24, 25]. Overall, people with high monthly gambling expenditure relative to net income, and men in particular, are more likely to be socio-economically vulnerable individuals [26].

Gambling frequency is typically assessed by asking people how many times they have engaged in gambling within a certain period of time, or by asking their average frequency of participation within a certain time frame [18]. A high frequency of gambling, participation in multiple game types and high gambling severity are associated with high total expenditure [27–29]. Although high gambling frequency is associated with gambling harms, only some frequent gamblers experience harm [30]. On the other hand, even occasional gamblers may experience harm [11, 13, 31].

There are various forms of gambling and their nature varies [32]. A simple classification distinguishes between lottery-style and wagering-style games. Another classification is based on game provider [18]. Finland is one of the countries where games are provided by a government regulated monopoly, although non-monopoly games are available online. Game types can also be classified based on means of access, such as direct face-to-face gambling or remote access [18]. Another access-based classification distinguishes between online and land-based games [32]. Furthermore, game types are classified based on whether their outcome is determined by chance, skill or a combination of chance and skill [33]. Games such as slot machines, lotteries, scratch cards, bingos, roulettes and dice games are fundamentally chance-based games, whereas poker and blackjack, for instance, also include elements of skill [34]. Another way to categorize game types is to look at their structural characteristics, which are event frequency, event duration, bet frequency and pay out interval [5]. In population studies, a common way of inquiring about participation in different gambling types is to use a list of available game types [18].

There is gender differences in game type preferences: men tend to favour skill-based games, whereas women prefer games of chance [35]. Game type preferences were highly gendered in Sweden, although men in Sweden have decreased their participation in games of strategy and increased participation in games of chance in public spaces [36]. In any assessment of gambling participation, it is therefore important to consider both the number of different game types played and the frequency of gambling [7, 9, 10, 46]. Playing multiple game types is associated with online gambling, and among females in particular online gambling may be related to higher gambling expenditure and at-risk and problem gambling [37].

In 2015, 23% of Finns gambled only one game type, predominantly weekly lottery games [20]. It is beneficial to take a broader view on gambling participation and also consider overall gambling frequency, gambling mode and number of game types gambled. Furthermore, an examination of different game types played by active gamblers and more occasional gamblers is a novel way of studying patterns of gambling expenditure and relative expenditure concurrently.

## Methods

### Aim, design and setting

This cross-sectional study aims to measure gambling expenditure by game type while controlling for demographics and other gambling participation factors, such as gambling frequency, number of game types played and gambling mode. We used two measures of gambling expenditure: weekly gambling expenditure and gambling expenditure in relation to net income. A further aim was to find out how each game type is associated with gambling expenditure when the number of game types played is adjusted for.

Until December 2016, Finland had a three-way monopoly system (Veikkaus, Finland’s Slot Machine Association [FSMA] and Fintoto) in which each game provider had the right to offer gambling services [38]. In 2017, these service operators were merged into a single company.

### Data collection

The data were drawn from the Finnish Gambling 2015 survey [20]. A random sample of 7400 persons aged 15–74 whose mother tongue was Finnish or Swedish and who resided in mainland Finland were approached by Statistics Finland. In total 4515 computer-assisted telephone interviews lasting on average 18 min were completed. The study was described to the potential participants as a survey about ‘gambling and opinions about gambling’. The response rate was 62% (men 62%; women 61%). Attrition is described in more detail elsewhere [20, 39].

The data were weighted based on gender, age and region of residence. Respondents who were allowed to gamble legally (≥ 18 years) and who had gambled during the past year were included in the study (*n* = 3555).

### Gambling expenditure

Gambling expenditure (GE) was inquired with the question: ‘Roughly how much money do you spend on gambling in a typical week (€)?’. If the respondent did not gamble each week, the interviewer was instructed to advise the respondent to give an estimate of their spending when they did gamble. In this study, GE was examined using two measures: weekly gambling expenditure in euros (€) and relative gambling expenditure (%).

Weekly gambling expenditure (WGE) was rescaled if respondents indicated an overall gambling frequency of less than once a week using the formula WGE = F*GE/365.25*7 [7–8], where

- F = 30 if past-year gambling frequency was 2–3 times a month
- F = 12 if past-year gambling frequency was once a month
- F = 6 if past-year gambling frequency was less than monthly

For weekly gamblers WGE = GE.

Relative gambling expenditure (RGE) was calculated using WGE and 2014 register data on personal net income provided by Statistics Finland. Personal net income consisted of total gross income (wages and salaries, investment income, benefits and allowances) minus taxes. The relative expenditure measure was formed by estimating yearly expenditure (WGE/7*365.25) and dividing it by personal net income. RGE thus represented the percentage of income used on gambling. For 361 participants it was not possible to calculate relative expenditure either because their net income was 0 euros (*n* = 12), or they did not report their gambling expenditure (*n* = 353).

### Gambling participation

Participants were asked whether they had gambled on 18 predefined game types during the past 12 months (yes/no). These game types were recoded into 12 game types because of the small size of groups among certain game types and to limit the number of variables added to the model. The recoded game types were: weekly lottery games, fast-paced daily lottery games (such as instant e-lotteries and e-Bingo), low-paced daily lottery games (such as Keno), scratch cards, betting games (including betting several teams at once, fixed odds betting, correct score and live betting) and casino games (live casino games in a casino or table games, such as roulette or Black jack run by a croupier outside a casino). Game types also included slot machines, horse race betting and private betting. Online poker included poker on the FSMA website; on the website of a private gaming company Ålands Penningautomatförening (PAF), while non-poker games on the FSMA online casino were treated as a separate game type. Finally, non-monopoly gambling included non-poker gambling outside the Finnish monopoly system, including non-monopoly and PAF games both online and on ferries between Finland, Estonia and Sweden.

Then, the number of game types played was calculated and recoded into four categories, since the association between gambling expenditure and number of game types was not linear. Also, we wanted to have estimates for different numbers of game types instead of only one estimate for a continuous variable. A cutoff of four or more games types was used to create roughly equal sized groups. Furthermore, there was a clear increase in the proportion of problem gamblers between gamblers with three and four game types (3.9 and 7.4%), and this cutoff point has been associated with problem gambling [20]. Overall gambling frequency was calculated based on the game type in which the gambler was most active. Then, gambling frequency was also recoded: at least once a week, 1 to 3 times a month and less often than once a month.

Following the example of previous studies [40, 41], gambling mode was classified as online gambling if the person had gambled online during the past year. Online gamblers included gamblers who may have participated in land-based gambling. The rest of the responses were classified as land-based gambling only.

### Weekly gambling

Game types were categorized by distinguishing active gamblers (‘at least once a week’) and more occasional gamblers (‘less than once a week’) and ‘non-players. This classification was used to assess the added effect of frequent gambling on 12 game types on gambling expenditure when controlling for overall gambling frequency.

### Data analysis

Two separate multiple log-linear regression models were used to explain the variation of WGE and RGE, since the distributions of both dependent variables were skewed to the right. In both models the independent variables were gender, age group, education level (demographic variables), overall gambling frequency, number of game types played and gambling mode (participation factors). Additionally, the nine game types were entered into the models using a stepwise forward method to find out which specific game types contributed to explaining WGE and RGE after controlling for demographics and participation factors. Casino games, non-poker games on the FSMA online casino and private gambling were excluded before stepwise regression because of the small group size of weekly gamblers. Exponentiations of beta coefficients (exp(β)) were interpreted as percentage differences between a subcategory and a reference category. WGE and RGE means were calculated separately for each of the nine game types by gambling frequency, and means were presented in two figures for the whole data and by number of game types (1–2 game types vs. at least three game types). If there were less than three respondents in a subcategory the corresponding mean was rounded to lower disclosure risk. All analyses were weighted based on gender, age and region of residence. Log-linear regression analysis was conducted using SPSS version 23.0 and the mean figures were constructed using R [42].

## Results

### Demographics

Nearly half (46.2%) of the 3555 respondents were women (Table 1). The respondents’ mean age was 48.38 years. Most participants had basic vocational qualifications (33.4%) or a higher degree (42.9%).

**Table 1:** Demographics and factors related to gambling participation

	%	N
Gender		
Woman	46.2	1644
Man	53.8	1911
Age group		
18–24	9.1	325
25–34	14.9	529
35–44	15.8	563
45–54	18.3	649
55–64	22.7	806
65–74	19.2	683
Education level		
Up to lower secondary education	15.2	542
Upper secondary	7.9	281
Basic vocational qualification	33.4	1188
Short cycle tertiary education	16.6	591
Bachelor’s or equivalent	14.9	530
Master’s or equivalent	11.4	407
Missing	0.5	16
Overall gambling frequency		
Less often than monthly	27.5	979
1 to 3 times a month	27.4	975
Weekly or more often	45.0	1600
Number of game types		
1	29.6	1051
2	26.8	953
3	17.3	616
4 or more	26.3	935
Gambling mode		
Strictly land-based	71.4	2539
Online	28.69	1016
Total	100	3555

Weighted based on gender, age and region of residence (N = 3555, non-weighted)

### Weekly gambling

The different game types differed in popularity (Table 2). More than one-third (37.8%) of the gamblers played lottery games on a weekly basis. The second most common game types played on a weekly basis were low-paced daily lottery games (9.3%), slot machines (7.1%) and betting games (5.0%).

**Table 2:** Weekly gambling by game types

	**%**	**N**
Weekly lottery games^a^		
non-player	11.5	408
less than once a week	50.7	1802
at least once a week	37.8	1344
Fast-paced daily lottery games^a^		
non-player	92.3	3282
less than once a week	7.1	252
at least once a week	0.6	21
Low-paced daily lottery games^a^		
non-player	72.2	2566
less than once a week	18.4	655
at least once a week	9.3	332
Scratch cards^a^		
non-player	47.3	1680
less than once a week	50.8	1805
at least once a week	2.0	70
Betting games^a^		
non-player	82.3	2925
less than once a week	12.7	452
at least once a week	5.0	178
Casino games^a^		
non-player	91.8	3265
less than once a week	8.0	285
at least once a week	0.1	5
Slot machines^a^		
non-player	65.0	2309
less than once a week	27.9	993
at least once a week	7.1	253
Horse games^a^		
non-player	93.0	3306
less than once a week	5.6	200
at least once a week	1.4	49
Private betting		
non-player	95.2	3383
less than once a week	4.7	168
at least once a week	0.1	4
Online poker^a,b,c^		
non-player	96.5	3430
less than once a week	3.0	106
at least once a week	0.5	19
Non-poker games on FSMA online casino^a^		
non-player	98.1	3489
less than once a week	1.7	60
at least once a week	0.2	6
Non-monopoly gambling^b,c^		
non-player	85.7	3046
less than once a week	13.6	484
at least once a week	0.7	25
Total	100	3555

Weighted based on gender, age and region of residence (N = 3555, non-weighted). ^a^Finnish gambling monopoly games; ^b^PAF, Ålands Penningautomatförening’s games; ^c^Gambling internationally outside the Finnish gambling monopoly. *FSMA* Finland’s Slot Machine Association

### Models explaining WGE and RGE

WGE was available for 3202 respondents and averaged 9.71 €/week (SD 43.72). RGE was available for 3194 respondents and averaged 3.0% of personal net income (SD 12.73). Using the stepwise forward method, eight game type variables were included in the models; only fast-paced lottery games were excluded. The models explained the higher amount of weekly expenditure (χ2 (33) = 3716.19, *p* < .001) and relative gambling expenditure (χ2 (33) = 3314.94, p < .001) statistically significantly (Table 3). Males’ weekly spending was 39% higher than females’, and relative to their annual net income 22% higher than females’ spending. Age also had an effect on both expenditure measures. Almost all age groups spent less on gambling than persons aged 65–74. Relative to personal net income, however, gamblers under 25 spent 79% more than those aged 65–74. The effect of education level on both expenditure measures was reversed as almost all education groups spent more on gambling than those with the highest education level (Master’s or equivalent). Relative to their personal net income, those who had a lower secondary education or less spent nearly three times more than their highly educated counterparts.

**Table 3:** Multiple log-linear regression models explaining weekly and relative gambling expenditure

	Weekly gambling expenditure (€)		Relative gambling expenditure (%)	
	exp(β)	95% CI	exp(β)	CI 95% CI
Gender				
Female	1.0		1.0	
Male	1.39***	1.28–1.50	1.22***	1.12–1.34
Age group				
65–74	1.0		1.0	
55–64	0.97	0.87–1.08	0.89	0.79–1.01
45–54	0.90	0.80–1.01	0.77***	0.67–0.88
35–44	0.87*	0.76–0.99	0.76***	0.65–0.88
25–34	0.74***	0.64–0.85	0.86	0.73–1.01
18–24	0.69***	0.59–0.82	1.79***	1.48–2.16
Education level				
Master’s or equivalent	1.0		1.0	
Bachelor’s or equivalent	1.32***	1.14–1.53	1.66***	1.41–1.95
Short cycle tertiary education	1.40***	1.21–1.61	1.96***	1.67–2.30
Basic vocational qualification	1.48***	1.30–1.68	2.20***	1.90–2.54
Upper secondary	1.17	0.98–1.40	1.97***	1.61–2.40
Up to lower secondary education	1.48***	1.28–1.72	2.88***	2.43–3.41
Overall gambling frequency				
Rarely than monthly	1.0		1.0	
1–3 times a month	4.68***	4.21–5.20	5.00***	4.44–5.63
Once a week or more	14.20***	11.91–16.93	16.22***	13.29–19.78
Number of game types				
1	1.0		1.0	
2	1.06	0.93–1.19	1.07	0.93–1.23
3	1.24*	1.05–1.47	1.32**	1.08–1.60
4 or more	1.52**	1.19–1.94	1.62**	1.23–2.13
Gambling mode				
Strictly land-based	1.0		1.0	
Online	1.10*	1.00–1.20	1.01	0.92–1.12
Weekly lottery games				
Non-player	1.0		1.0	
Less than once a week	0.88	0.76–1.01	0.67***	0.56–0.79
At least once a week	1.17	0.97–1.42	0.83	0.67–1.03
Low-paced daily lottery games				
Non-player	1.0		1.0	
Less than once a week	1.08	0.97–1.21	1.03	0.91–1.17
At least once a week	1.67***	1.44–1.93	1.62***	1.37–1.91
Scratch games				
Non-player	1.0		1.0	
Less than once a week	0.99	0.89–1.10	0.91	0.81–1.03
At least once a week	1.79***	1.37–2.33	1.44*	1.07–1.94
Betting games				
Non-player	1.0		1.0	
Less than once a week	1.20**	1.05–1.36	1.16*	1.01–1.34
At least once a week	1.78***	1.49–2.12	1.80***	1.47–2.20
Slot machines				
Non-player	1.0		1.0	
Less than once a week	0.84**	0.75–0.95	0.83**	0.73–0.94
At least once a week	1.31**	1.10–1.56	1.43***	1.18–1.74
Horse games				
non-player	1.0		1.0	
less than once a week	1.09	0.93–1.27	1.08	0.91–1.30
at least once a week	2.46***	1.82–3.31	2.55***	1.82–3.57
Online poker				
non-player	1.0		1.0	
less than once a week	1.27*	1.03–1.58	1.24	0.97–1.58
at least once a week	1.83*	1.13–2.97	1.79*	1.03–3.09
Non-monopoly gambling				
non-player	1.0		1.0	
less than once a week	1.06	0.94–1.19	1.03	0.89–1.18
at least once a week	4.09***	2.68–6.25	3.59***	2.23–5.79

Weighted based on gender, age and region of residence (*N* = 3202 in WGE model and *N* = 3194 in RGE model). Significance probabilities * *p* < .05; ** *p* < .01; *** *p* < .001

All participation factors had an effect on expenditure. Those who gambled once a week or more spent 14 times more than those who only gambled rarely and 16 times more relative to their personal net income. Engaging in four or more game types increased weekly expenditure and relative expenditure by 52 and 62%, respectively, compared to those who played one game type. Gambling online increased weekly expenditure by just 10% and was not statistically significantly associated with relative gambling expenditure.

Those who played non-monopoly games at least once a week had a four times higher expenditure and a three-and-a-half times higher relative expenditure than gamblers who did not play abroad. Other game types where weekly gambling had an effect on expenditure measures were low-paced daily lottery games, scratch games, betting games, slot machines, horse race betting and online poker, where weekly gamblers had a 31–155% higher expenditure than the corresponding non-players.

### WGE and RGE by game types

**Fig. 1:**
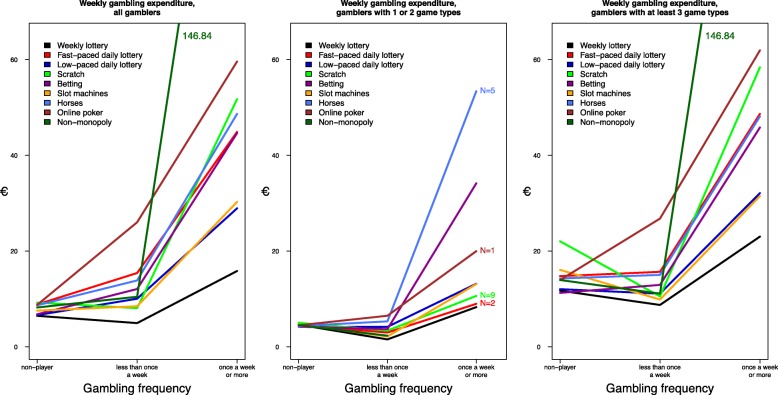
Mean weekly gambling expenditure (euros) by game type (all, 1–2 game types, ≥ 3 game types)

**Fig. 2:**
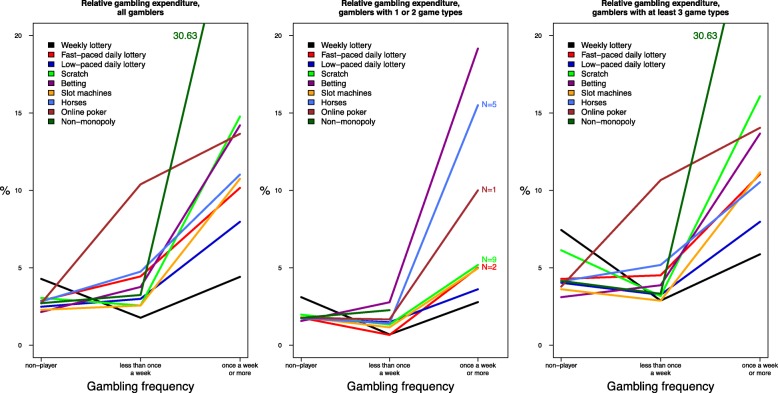
Mean relative gambling expenditure (%) by game type (all, 1–2 game types, ≥ 3 game types)

Those who played non-monopoly games had the highest mean WGE (146.84 €/week) among weekly gamblers (Fig. 1). Other game types with high mean WGE were online poker (59.61 €/week), scratch games (51.77 €/week) and horse race betting (48.67 €/week). RGE means were highest among those who gambled weekly non-monopoly games (30.63%), scratch games (14.77%), betting games (14.20%) and online poker (13.65%) (Fig. 2).

Fast-paced daily lottery games (*n* = 2), scratch games (*n* = 9), horse race betting (*n* = 5), online poker (*n* = 1) and non-monopoly gambling (*n* = 0) had less than 10 weekly gamblers who gambled only one or two game types (Figs. 1-2). Among those who gambled only one or two game types, the highest WGE and RGE means were recorded for horse race betting and other betting games. WGE and RGE means were lower for those who played one or two game types compared to the corresponding means for all gamblers, except for horse race betting (WGE means 53.40 €/week vs. 48.67 €/week and RGE means 15.50% vs. 11.02%) and betting games (RGE means 19.16% vs. 14.20%). The WGE and RGE means for those who played at least three game types weekly were similar to the corresponding means for all gamblers.

## Discussion

Male gender, lower education level, higher gambling frequency and higher number of game types increased both WGE and RGE, which is in line with previous research (e.g. [20–22]). Our results also indicated that younger age decreased WGE, but increased RGE. This may be partly explained by lower overall income, since low income in general [15, 16] is a risk factor for excessive gambling it can be seen as possibly posing greater harm to this specific age group, such as indebtedness that may in turn increase harmful gambling.

Overall gambling frequency was the strongest explanatory factor of both WGE and RGE, which supports the results of previous research [9]. Weekly horse race betting, non-monopoly gambling and online poker had the greatest increasing effect on expenditure, but scratch games, betting games and daily low-paced lottery games also contributed significantly to overall expenditure. Furthermore, betting games and online poker were associated with higher expenditure even when they were played less often than weekly. Our results suggest, in certain circumstances high WGE on these particular game types may be seen as indicators of unhealthy gambling involvement, as has been previously suggested (e.g.[9, 10]).

Some studies indicate, that sports betting is associated with problem gambling [8, 45] while some studies indicate that it is not [7, 10]. Sports betting and poker can be viewed as a lifestyle practice, often a regular feature of social interaction and leisure time [43]. Gamblers may be inclined to take unnecessary risks to demonstrate their “knowledge” of the game and enhance their social esteem. The unique feature of sports betting and poker is competition [43]. Game providers should avoid targeting this group with advertisements that create false notions of expertise [27]. Furthermore, a recent Australian study examined the risk factors for low risk gambling, moderate risk gambling and problem gambling amongst sports betters [44]. Their results indicate, that gambling expenditure, number of accounts with different operators, number of different types of promotions used and gambler’s impulsiveness were significantly higher for all above mentioned risk groups, while age, gender, some normative factors, and particular sports betting variables only applied to those with the highest level of gambling-related problems. These results suggest, that when assessing risk factors for problematic gambling, severity of gambling should be taken into account, when possible, thus different levels of gambling problems should be assessed separately when possible.

Weekly gambling outside the Finnish gambling monopoly had the greatest increasing effect on gambling expenditure. This result must be interpreted with caution, however, since there was only a small number of weekly non-monopoly gamblers. Non-monopoly gamblers tend to be heavy consumers of several game types [5, 6, 45], including monopoly games. In addition, non-monopoly gambling remains as a somewhat indefinite game type category, since it may, in fact, include any number of game types, as well as, any number of player accounts with different international gaming operators. Therefore, it may represent merely a time spent on gambling rather than a certain game type.

Frequent playing of several games is associated with gambling problems [7, 9, 10]. In addition, online problem gamblers are often mixed-mode gamblers who play multiple types of games [47–49]. The major justification for the Finnish gambling monopoly is that it has the potential to reduce gambling harms, and the updated Lotteries Act furthermore places emphasis on prevention [38]. As in many other countries, Finnish gambling operators have in recent years been working to develop tools for responsible gambling (RG). Recent Finnish surveys indicate that RG tools are used quite rarely and that gamblers’ awareness about these tools must be improved [11, 12].

One of the games that increased gambling expenditure was weekly horse race betting. Based on register data provided by gambling operators, a typical gambler is a middle-age man who gambles seven times a month, spending on average 33 euros a day when gambling [22]. In Finland, online horse race betting seems to be concentrated: most gamblers spend rather small amounts of money, but there is a small group of active bettors who contribute a large proportion of total turnover [22]. Participation and interest in horse racing and betting seems to be a social cross-generational process [50], which is not the case with other types of betting. LaPlante and colleagues studied gambling problems, type of gambling and gambling involvement and noticed that the relationship between both horse race betting and private betting and problem gambling changed when gambling involvement factors were adjusted for [9]. In other words, gambling only these two particular betting games seemed to protect gamblers from problems [7, 9]. In fact, they suggest that engaging in game types including peers might encourage control and preclude excessive gambling [9], which is opposite to the findings for sports betting [43].

Weekly lottery games were not associated with high expenditure, but daily lottery games were. Weekly lottery games are slow pace and sometimes perceived as a ‘soft’ type of game [4], or indeed not even viewed as a form of gambling at all [51]. Nevertheless, there are some addictive features of lottery games that are salient to the psychology of lottery gambling [52]. Recent developments of lottery games have extended gambling frequency from weekly to biweekly and daily gambling, but also changed their geography from regional or national to transnational and gambling mode from land-based to online platforms. These changes have increased the addictiveness of this game type. We suggest that future studies should make a clear distinction between different types of lottery games.

Finland has one of the highest per capita numbers of slot machines in Europe. In our model, weekly slot machine gambling was also associated with higher expenditure, but it was not among the most significant game types. Slot machine gambling is nevertheless associated with gambling-related harms [7, 8, 38, 43, 45]. Moreover, based on the national helpline Peluuri, the primary game types that cause problems among Finnish gamblers are EGMs (69%), betting games (9%), poker (5%) and casino games (5%) [53]. On the other hand, some studies indicate that slot machines are not among the top five game types associated with problem gambling [4, 10].

The results provide useful information about gambling expenditure patterns by game type. At the same time, they underscore the fact that gambling participation needs to be studied in its entirety. This was particularly clear in Figs. 1, 2, which showed that for those gambling at least three game types weekly, WGE and RGE means were similar to the corresponding means for all gamblers. Gambler profiles can be grouped based on gambling participation and the combination of different game types played [46, 54]. A study on gambling clusters indicates that gambling on slot machines, sports betting and playing multiple games are the strongest indicators of gambling problems [46]. These clusters provide useful leads for future studies on game types and gambling expenditure.

There is evidence that high gambling expenditure is associated with gambling-related harms [18, 31, 46, 55, 56]. However, we still have an incomplete picture of what level of expenditure indicates harms. A Finnish study that used the South Oaks Gambling Screen [57] indicates that on average, problem gamblers spend 11.8%, probable pathological gamblers spend 17.3%, and non-problem gamblers spend 1.6% of their monthly net income on gambling [15]. Gender differences have also been reported in the relative amount of income associated with problematic gambling in Finland [16].

### Study limitations

Phrasing of the question and response instructions matter when inquiring about gambling expenditure [58, 59]. In our study, was inquired by one question instead of assessing it separately for each game types, and gambling expenditure was not explained in the instructions for the respondents. Furthermore, game types were inquired using a list of available game types provided by different operators. These gaming providers have their own RG tools, but we were unable control for the use of these tools. Furthermore, the number of games varies in different game type categories. Moreover, specific game types may be played more frequently than others due to the nature of the games [60]. For example, it is quite rare that live casino games are played on a weekly basis. In our study, however, live casino games included table games such as roulette and Blackjack run by a croupier outside a casino. PAF games on cruise ferries are rarely, if ever, played on a weekly basis. Weekly non-monopoly gambling therefore mainly reflect non-monopoly online gambling. The game type list which includes several gambling modes and game characteristics can create overlapping categories [18]. Furthermore, there is the possibility of incomplete coverage, meaning that some game types are assessed by subtypes and others are not [18]. For example, betting games were divided into horse games and other types of betting, and three subtypes of lottery games were identified.

Overall, gamblers frequently underestimate their losses [59, 61–65]. Despite this, it has been shown that self-reported gambling losses correlate with register-based losses. Gamblers with higher losses, however, tend to have more difficulty estimating their gambling expenditure [64, 65]. A high intensity of play, problem gambling and the type of game gambled may also cause estimation bias. People who play games that carry a social stigma (such as EGMs) may underestimate their expenditure [59]. Furthermore, self-reported losses have proved to be more accurate when using a 3-month rather than a 12-month time frame [64]. Our results therefore give an estimation of overall expenditure. One of strengths of this study is its high response rate.

## Conclusions

Gambling frequency was the strongest indicator of high expenditure, as also suggested in previous studies [7, 9]. However, this study provides some useful information about gambling expenditure patterns by game type. Different game types had different effect sizes on gambling expenditure, and we identified several games types that increase both WGE and RGE. Weekly gambling on horse races and non-monopoly games had the greatest increasing effect on expenditure. Great effort should be made by game providers and policy makers to inform individuals about these particular games and possible harms related to them. In addition, betting games, sports betting and online poker in particular, were associated with higher expenditure even when they were played less often than weekly. Similarly, more active harm-minimizing initiatives are recommended particularly for sports bettors and online poker players. However, different game types also varied according to their popularity, and therefore the extent of potential harms caused by high expenditure also varies on the population level. Studies of gambling problems have found that few gamblers play at high-risk levels, but large proportions gamble at low-risk levels [31, 66, 67]. Therefore, in order to prevent and reduce gambling-related harms, lower risk gamblers should also be targeted in preventive actions. Based on our results, future prevention and harm minimization efforts should be tailored to different game types for greater effectiveness.

## Abbreviations

EGM: Electronic Gaming Machine
FSMA: Finland’s Slot Machine Association
PAF: Ålands Penningautomatförening
RGE: Relative gambling expenditure
WGE: Weekly gambling expenditure
